# AI-Driven Robotic PCI: A Perception-to-Action Framework for Coronary Guidewire Control

**DOI:** 10.3390/bioengineering13080848

**Published:** 2026-07-23

**Authors:** Zijing Liu, Huanming Xu, Zhendong Liu, Jun Ma, Jian Liu, Pengfei Bi, Liming Huo, Xiaofeng Su, Bo Yu, Jingbo Hou, Li Yin, Lei Wang, Fucang Jia, Shoujun Zhou, Jing Yang, Guangyao Zhai

**Affiliations:** 1 Department of Cardiology, Beijing Luhe Hospital Affiliated to Capital Medical University, No. 82 Xinhua South Road, Tongzhou District, Beijing 101149, China; lhyylzj@163.com (Z.L.);; 2 Raysight Medical Inc., Room 1201, Overseas Students Venture Building Phase II, Nanshan District, Shenzhen 518057, China; huanming.xu@raysightmed.com (H.X.);; 3 Department of Cardiology, Peking University People’s Hospital, No. 11 Xizhimen South Street, Xicheng District, Beijing 100044, China; 4 Department of Cardiology, The Second Affiliated Hospital of Harbin Medical University, No. 246 Xuefu Road, Nangang District, Harbin 150086, China; 5 Shenzhen Institutes of Advanced Technology, Chinese Academy of Sciences, No. 1068 Xueyuan Avenue, Shenzhen University Town, Nanshan District, Shenzhen 518055, China

**Keywords:** AI-driven robotic PCI, biomedical engineering, translational bioengineering, robotic guidewire control, fluoroscopic perception, dynamic coronary mapping, vessel-coordinate state estimation, preclinical validation

## Abstract

Robotic percutaneous coronary intervention (PCI) remains predominantly teleoperated, while the most demanding part of the procedure, guidewire navigation through a moving coronary tree under fluoroscopy, still depends on the continuous human interpretation of vessel anatomy, cardiac phase, guidewire position, and target location. We present a preclinical perception-to-action framework for AI-driven robotic PCI that integrates fluoroscopic perception, dynamic coronary vessel memory, vessel-coordinate state estimation, and robot-executable guidewire command generation on a robotic PCI platform. During contrast angiography, phase-indexed vessel–catheter templates and a dynamic vessel-coordinate coronary map are generated. During guidewire manipulation without contrast injection, live fluoroscopy is segmented into catheter and guidewire structures, cardiac phase is estimated by overlap between the live catheter–guidewire skeleton and stored vessel–catheter templates, the guidewire is assigned to the most likely vessel branch, and the distal tip is projected to a vessel-coordinate target representation for robotic action generation. The segmentation dataset contained 6269/1567/957 vessel images, 3857/964/537 guidewire images, and 4796/1199/667 catheter images for training/validation/test splits, respectively. Test-set Dice scores were 90.7% for vessels, 92.6% for catheters, and 89.0% for guidewires. In 623 real-time fluoroscopy frames from physician-supervised animal experiments, phase selection accuracy was 589/623 (94.6%; 95% CI, 92.5–96.1%) and vessel assignment accuracy was 575/623 (92.3%; 95% CI, 89.9–94.1%). Across 34 scenario-level episodes and their associated command-level decisions, correct-command rates ranged from 83.6% to 95.6% across target navigation and safety scenarios. These results provide preclinical evidence that live fluoroscopic perception can be converted into robot-executable coronary guidewire actions within an integrated AI-robotic PCI workflow. The study was designed to establish early system feasibility rather than to prove clinical superiority, large-scale generalization, or comparative advantages over manual or teleoperated robotic PCI.

## 1. Introduction

Robotic intervention is increasingly moving beyond pure teleoperation toward systems that couple perception, task representation, and machine-executable action [1,2,3,4]. This transition is particularly compelling in vascular intervention, where procedural decision making depends on the continuous interpretation of fluoroscopic images, anatomical context, and device behavior. In percutaneous coronary intervention (PCI), guidewire navigation through a deforming coronary tree remains one of the most technically demanding steps because vessel visualization is intermittent, the anatomy is dynamic, and the wire must be directed through branching structures under limited visual information.

Contemporary robotic PCI platforms have established the feasibility of remote intracoronary device manipulation and have demonstrated potential ergonomic and radiation-related benefits for operators [5,6,7,8,9]. However, current robotic PCI remains largely teleoperated. The robotic system executes physician commands, whereas the physician remains responsible for interpreting contrast angiography, inferring vessel position during non-contrast fluoroscopy, recognizing wrong-branch entry, and choosing the next guidewire action. From a biomedical engineering perspective, this leaves a substantial gap between robot actuation capability and image-driven procedural intelligence.

Dynamic coronary roadmapping addresses part of this gap by carrying coronary anatomical information from contrast angiography into subsequent fluoroscopic guidance [10,11,12]. Ma et al. reported dynamic coronary roadmapping based on catheter-tip tracking and deep-learning-assisted Bayesian filtering in X-ray fluoroscopy [13]. These methods are valuable because they improve anatomical context during intervention, but most such systems remain physician-facing navigation aids. A robotic controller requires more than a visual overlay. It requires a structured state that encodes the current cardiac phase, the vessel containing the guidewire, the guidewire-tip position relative to a target, and the action implication of that state. In non-contrast guidewire navigation, exact framewise coronary vessel ground truth is not visible to either the operator or the algorithm; the clinically meaningful requirement is therefore a phase-consistent anatomical approximation that is sufficiently stable to support safe guidewire action.

Parallel developments in fluoroscopic AI perception have improved the segmentation and localization of guidewires, catheters, and related interventional devices. Attention-based guidewire segmentation, real-time guidewire tracking, sim-to-real transfer, and data-efficient augmentation strategies have been proposed to address the thin, low-contrast, deformable nature of guidewires in fluoroscopy [14,15,16,17]. Reinforcement learning, transformer-based predictive control, and broader embodied-intelligence frameworks have also been explored for robotic or robot-assisted endovascular guidewire navigation [18,19,20]. Low-level robotic motion planning is another important part of robotic execution, and minimum-jerk trajectory planning has been used to generate smooth motions for translational parallel manipulators [21]. However, actuator-level trajectory smoothing addresses a different layer of the robotic stack from image-derived procedural state estimation and task-level guidewire action selection.

Yet, directly comparable work on integrated AI-driven robotic PCI remains limited. Most prior studies focus on one part of the problem, such as device perception, physician-facing roadmapping, simulated or phantom guidewire control, teleoperated robot actuation, or low-level trajectory planning. In contrast, the present study asks whether contrast-stage vessel memory and live non-contrast fluoroscopic perception can be organized into a robot-actionable vessel-coordinate state that drives physical guidewire commands during preclinical PCI navigation.

Within this broader literature context, the present study is best understood as a translational biomedical engineering effort in AI-driven robotic intervention. Its primary contribution is system integration: connecting fluoroscopic perception, dynamic vessel memory, vessel-coordinate state estimation, and robot actuation in a live preclinical intervention workflow. The novelty is therefore not a larger static image-recognition benchmark, but the closed-loop conversion of image-derived guidewire and vessel state into robot-executable coronary guidewire actions. The individual algorithms are important enabling components, but the central question is whether their outputs can be organized into a robot-actionable state that supports guidewire commands on an interventional robotic platform.

**Figure 1:**
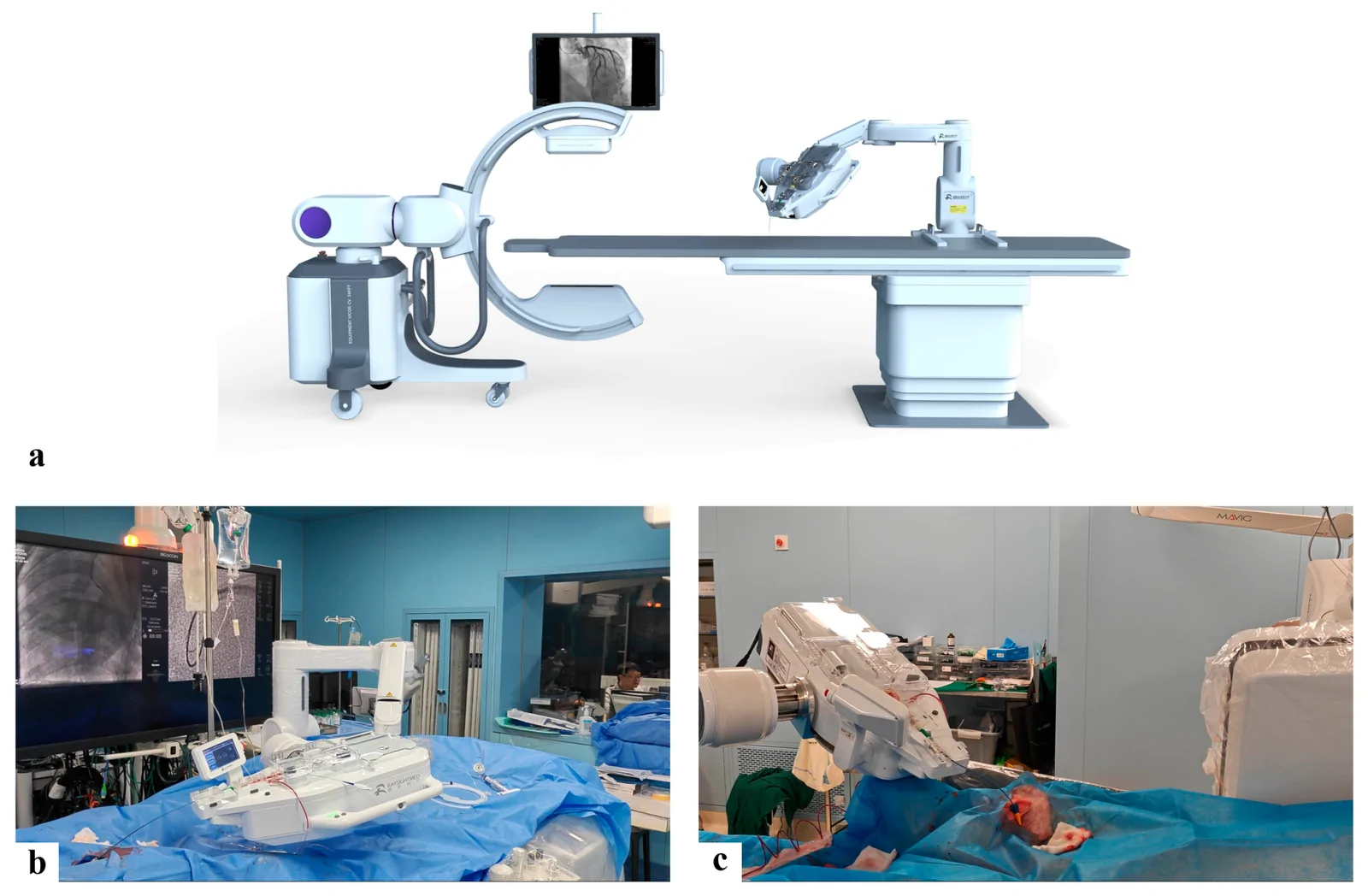
Robotic PCI platform and physician-supervised animal-experiment deployment. (**a**) High-level robotic PCI platform developed by Raysight Medical Inc. (Shenzhen, China), consisting of an operator console and bedside actuation unit for guiding catheter, guidewire, and stent/balloon delivery-device manipulation. (**b**,**c**) Physician-supervised animal-experiment deployment of the AI-driven robotic PCI workflow. In the AI-driven experiments reported here, the console–control interface was driven by the AI algorithm rather than by manual joystick operation.

**Figure 2:**
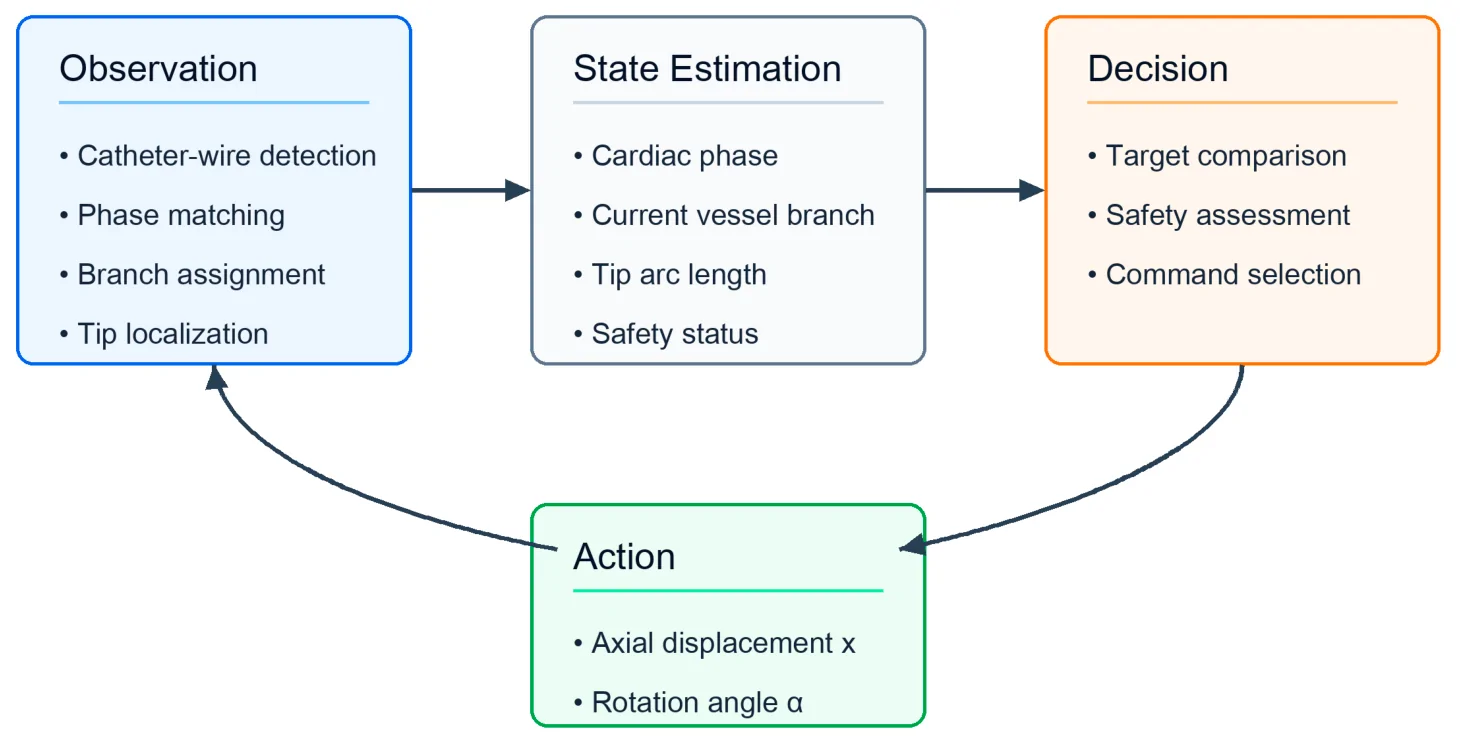
Closed-loop AI + robot control framework for PCI guidewire navigation. The system is organized as a recurrent decision–action–observation loop. The decision module receives the current vessel-coordinate state and the physician-defined target, then generates a PCI-relevant motion command. The robotic actuation module executes axial guidewire/catheter translation and rotational adjustment. The observation module analyzes live fluoroscopy to update guidewire detection, cardiac phase matching, vessel assignment, and target-relative position.

**Figure 3:**
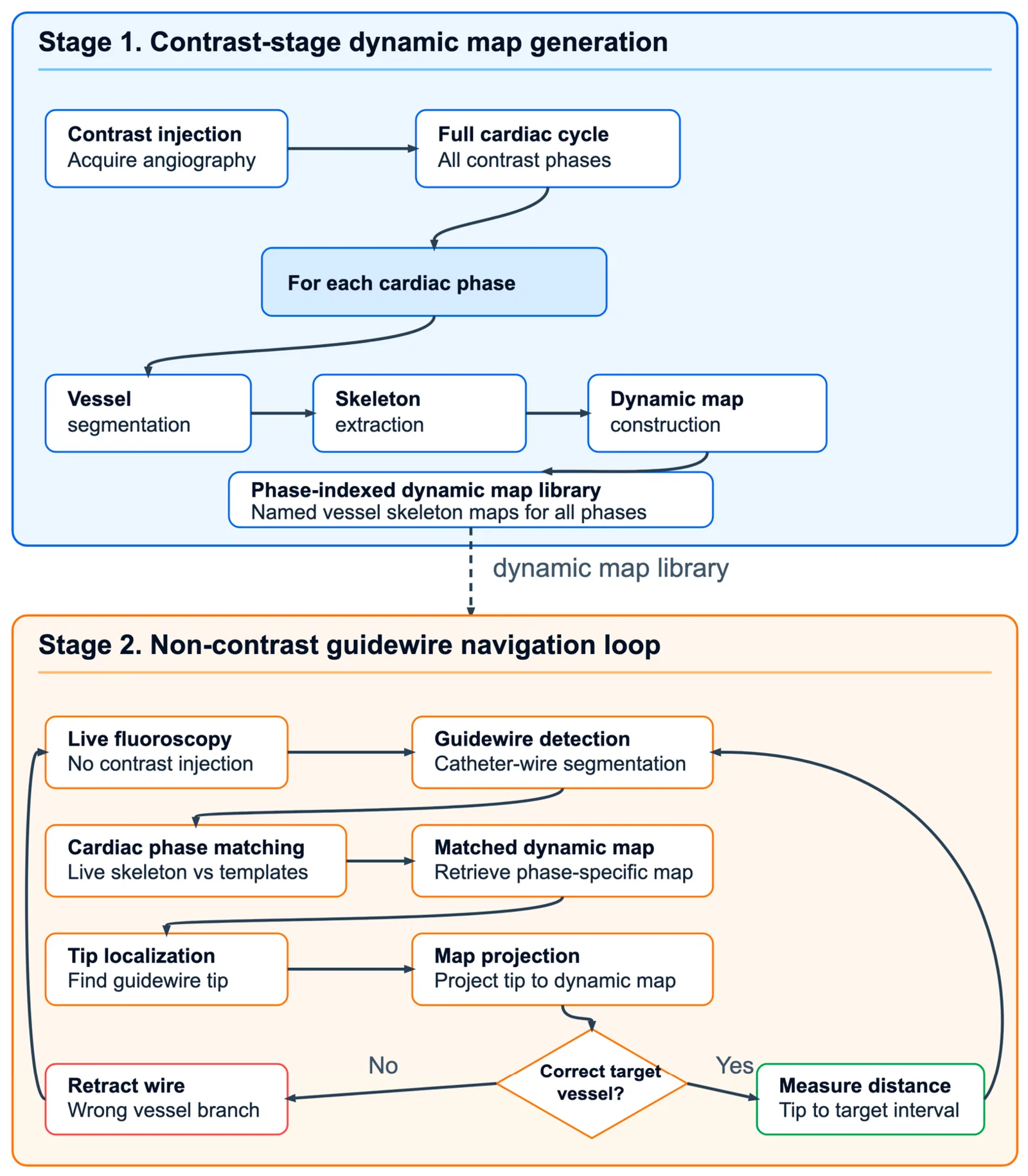
Two-stage workflow for dynamic coronary mapping and live robotic navigation. Stage 1: During contrast injection, a cardiac-cycle angiographic sequence is acquired, coronary vessels and the guiding catheter are segmented in each phase, vessel masks are skeletonized, and a phase-indexed dynamic coronary map library is generated. Stage 2: During guidewire navigation without contrast injection, the live catheter–guidewire structure is segmented and skeletonized, matched to the most likely cardiac phase, assigned to the corresponding dynamic vessel map, and projected to a guidewire-tip coordinate. The controller then decides whether to advance, retreat, stop, or rotate while advancing.

Here, we present a preclinical biomedical engineering framework for AI-driven robotic PCI guidewire control. The workflow was developed in physician-supervised animal experiments and integrates: (1) contrast-stage dynamic coronary map construction; (2) live non-contrast catheter–guidewire state estimation; (3) vessel-coordinate target representation; and (4) robot-command generation for guidewire advancement, retreat, stop, and rotation-assisted advancement. We evaluate the work as an integrated perception-to-action robotic workflow rather than as a standalone image-processing module. The robotic platform and experimental deployment are shown in Figure 1. The closed-loop control concept is summarized in Figure 2, and the two-stage procedural workflow is summarized in Figure 3.

## 2. Materials and Methods

### 2.1. Study Design and System Overview

This study evaluated an AI-driven robotic PCI workflow for coronary guidewire navigation in a physician-supervised preclinical animal-experiment setting. The methodology was organized around two sequential stages. In Stage 1, contrast angiography was used to construct a phase-indexed dynamic coronary map library that retained vessel topology and catheter geometry across the cardiac cycle. In Stage 2, guidewire manipulation was performed on non-contrast fluoroscopy, and live device perception was combined with the previously constructed map library to estimate a robot-executable vessel-coordinate state.

The robotic PCI platform used in this study was developed by Raysight Medical Inc. (Shenzhen, China). At a high level, the platform consists of an operator console and a bedside actuation unit. The console provides three control channels corresponding to guiding catheter manipulation, guidewire manipulation, and stent/balloon delivery-device manipulation, whereas the bedside unit performs the corresponding physical intracoronary device motions. Detailed mechanical, electrical, and low-level interface design information is proprietary and is outside the scope of this manuscript. In the experiments reported here, the console–control interface was driven by the AI algorithm rather than by manual joystick operation, thereby enabling live fluoroscopic interpretation to be translated directly into robot-executable guidewire commands.

Before the three physician-supervised animal experiments from which the quantitative results in this manuscript were derived, the workflow underwent iterative refinement through multiple additional animal experiments for research-and-development purposes. From a systems perspective, the framework decomposes robotic guidewire navigation into four connected layers: AI perception, dynamic vessel memory, vessel-coordinate state estimation, and robot action generation (Table 1). This decomposition reflects the fact that pixel-space guidewire detection alone is insufficient for robotic PCI. The robot must also determine anatomical context, phase context, target context, and procedural action relevance.

The evaluation was therefore designed as a system evidence chain rather than as a single-module benchmark. First, perception-module performance was measured using task-specific segmentation test sets. Second, vessel-coordinate state-estimation performance was measured on real-time fluoroscopy frames from animal experiments. Third, integrated command behavior was evaluated at the level of scenario episodes and sequential robot actions generated during physician-supervised navigation scenarios. These units are complementary but not interchangeable: image counts evaluate segmentation, frame counts evaluate real-time state estimation, scenario episodes evaluate task contexts, and command counts evaluate controller behavior within those episodes. This layered design avoids treating segmentation accuracy or frame number as a surrogate for robotic performance while still preserving the causal structure of the perception-to-action workflow.

### 2.2. Animal Preparation and Image Acquisition

All reported animal experiments were conducted at Bo’s (Guangzhou, China) Medical Technology Co., Ltd., a facility accredited by the China National Accreditation Service for Conformity Assessment (CNAS). The experimental protocol was reviewed and approved by the facility’s Institutional Animal Care and Use Committee. Animal preparation and anesthesia followed the approved institutional animal-experiment protocol and relevant Chinese animal-welfare standards. Three healthy Large White pigs (two males and one female, 12–18 months old, weighing 63–68 kg) confirmed to be free of major pathogens were selected for this study. This porcine model was used because its coronary access conditions, cardiac motion, and vascular intervention workflow provide a physiologically relevant preclinical setting for evaluating robotic guidewire navigation. Before the procedures, the animals were acclimated in a controlled environment maintained at 16–26 °C with a relative humidity of 30–70%.

Induction anesthesia was administered by intramuscular injection of a mixed regimen consisting of a Zoletil/tiletamine–zolazepam preparation at 1–1.5 mg/kg and xylazine hydrochloride at 1.5–2.5 mg/kg. After the animal became quiet, skin preparation was performed and an intravenous catheter was placed in an auricular vein. Anesthetic depth and consciousness were monitored during preparation. When deeper anesthesia was required, propofol at 0.5–1 mg/kg was administered slowly through the intravenous route. After complete loss of consciousness, endotracheal intubation was performed, the tube was positioned at an appropriate depth and fixed, and mechanical ventilation was initiated with respiratory parameters adjusted to the animal condition. Inhalational anesthesia was used for maintenance during the procedure.

Heart rate, blood pressure, oxygen saturation, and other vital signs were monitored throughout the experiment. Supportive vasoactive medications, including epinephrine, norepinephrine, and dopamine, were administered when clinically indicated to maintain physiologic stability.

Image acquisition followed a standardized protocol for the left anterior descending artery (LAD) and its major branches and for the left circumflex artery (LCX) and its major branches. Taking the LCX as an example, the guiding catheter was first engaged and its position was optimized to allow smooth guidewire passage into the LCX. The guidewire was then withdrawn to the catheter tip, and contrast medium was injected to acquire an angiographic sequence spanning at least one complete cardiac cycle with adequate vessel opacification; three consecutive cardiac cycles were recorded as the baseline angiogram. Subsequently, the catheter position was kept fixed, and the guidewire was advanced to the distal LCX, where contrast injection was performed to confirm wire location.

The guidewire was then retracted to the proximal LCX, and each major side branch was cannulated in anatomical order. At each branch, two contrast injections were administered, one at the ostium and one at the distal end, to verify guidewire position. After all major branches had been interrogated, the guidewire was withdrawn to the proximal LCX, and the branch-interrogation sequence was repeated twice to obtain complete datasets for the vessel. The LAD acquisition protocol followed the same logic. When switching between main vessels, the catheter could be re-engaged or repositioned; however, during the complete acquisition workflow for a single vessel, including repeated branch-interrogation sequences, the catheter position was kept unchanged whenever possible. After the completion of the experimental procedures, femoral artery compression was performed to achieve hemostasis, and the animals were returned to the housing facility for continued care according to the approved protocol.

### 2.3. Contrast-Stage Dynamic Coronary Map Construction

Let the contrast angiographic sequence be denoted by $I^{c}=\{I_{t}^{c}{\}}_{t=1}^{T}$, where t indexes cardiac phase. For each phase, the system estimated a coronary vessel mask $V_{t}$ and a guiding catheter mask $C_{t}$. The merged vessel–catheter template used for phase matching was defined as

(1) $$M_{t}=V_{t}\cup dilate(C_{t}).$$

This distinction between vessel-only and vessel–catheter representations is central to the workflow. Vessel masks were used to reconstruct the coronary tree, whereas merged templates preserved geometric context for later non-contrast-phase selection. These merged templates were operational phase-matching references rather than framewise anatomical ground truth. Standard post-processing, including connected-component filtering and morphological cleanup, was used to suppress segmentation artifacts before skeleton extraction.

Deep-learning perception modules were used as front-end segmentation components rather than as the controller itself. Coronary vessel and guidewire segmentation were based on nnU-Net-style models [22], and guiding catheter segmentation used an Attention U-Net-style architecture [23]. The segmentation models were selected as task-specific components based on development-stage performance and practical integration with the downstream mapping pipeline, rather than proposing a unified multi-task segmentation architecture. These models provided the vessel and device masks from which the downstream vessel-coordinate representations were constructed.

### 2.4. Reference Tree Extraction and Dynamic-Phase Propagation

A physician-selected contrast frame served as the static reference phase. The coronary vessel mask in this frame was skeletonized and converted into a named tree representation. The main branch was identified, bifurcation candidates were detected, and side branches were traced over the skeleton graph. Each named vessel v was represented as an ordered centerline $P_{s}(v)=\{p_{1},\ldots ,p_{n}\}$ with cumulative arc length $L_{s}(v)$.

The labeled reference tree was then propagated to the remaining cardiac phases. For each phase, the vessel mask was skeletonized, the main branch was extracted, and side branches were associated with the reference or neighboring phase by bifurcation proximity, branch order, and local path-shape similarity. When a branch was weakly visible or not confidently extracted, the previous phase branch path was locally translated to the estimated current bifurcation. This process yielded a dynamic coronary map library $G=\{G_{t}{\}}_{t=1}^{T}$ in which each phase-specific map retained named vessel centerlines.

**Figure 4:**
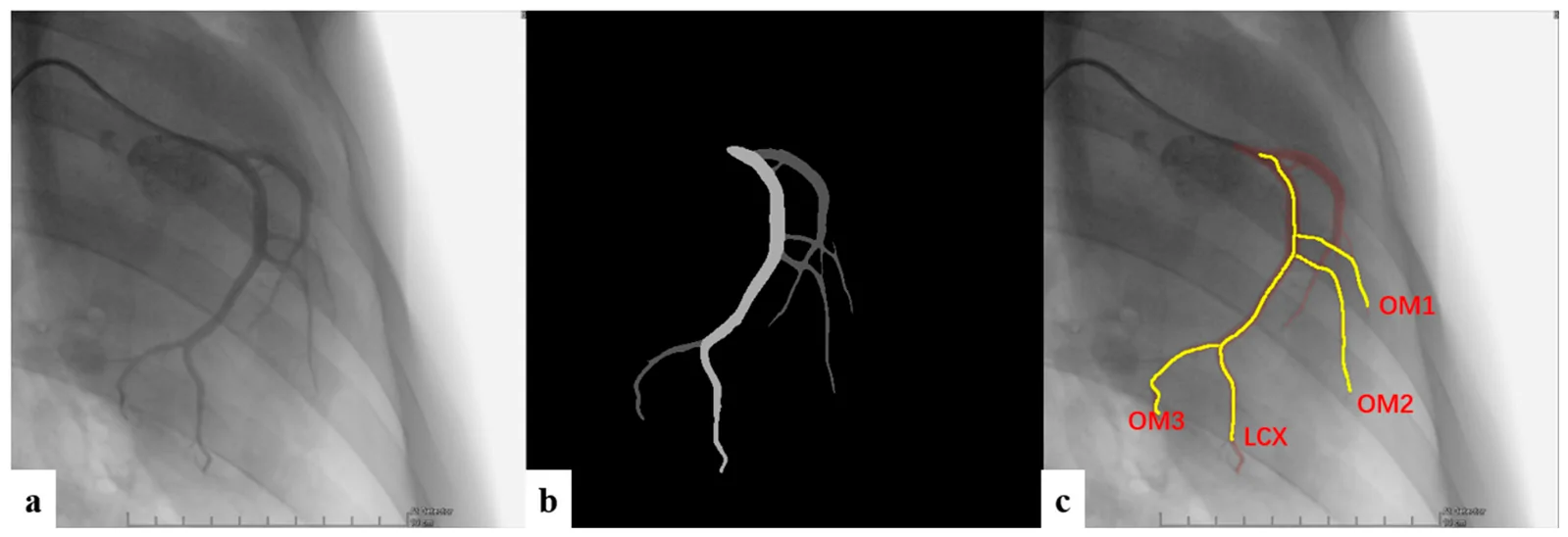
Dynamic coronary skeleton map generation from contrast angiography. (**a**) A contrast angiographic frame provides the visible coronary tree for the selected cardiac phase. (**b**) Vessel segmentation extracts the coronary foreground, followed by post-processing to remove small noisy components, fill holes, and suppress screen annotations; lighter gray indicates the segmented main branch, and darker gray indicates segmented side branches. (**c**) Skeleton and tree extraction convert the vessel mask into named centerlines by identifying the main branch, detecting bifurcations, tracing side branches, filtering anatomically implausible branches, and assigning branch names. The same tree representation is propagated across phases to form the dynamic map library used during non-contrast guidewire navigation.

The resulting library served as a phase-conditioned vessel-coordinate reference for downstream state estimation and robotic control. It should be interpreted as a functional guidance representation rather than as a fully independently validated frame-by-frame anatomical registration of every coronary branch across the cardiac cycle. This distinction is important because, during subsequent non-contrast guidewire navigation, the coronary lumen is no longer visible and exact dynamic centerline ground truth is not available. Figure 4 shows the contrast-stage dynamic map generation concept.

### 2.5. Vessel-Coordinate Target Representation

The physician-defined target was encoded as $y=(v^{⋆},r^{⋆})$, where $v^{⋆}$ denotes the target vessel and $r^{⋆}\in [0,1]$ denotes the relative arc-length position along that vessel in the reference map. The corresponding reference target arc length was

(2) $$x_{s}=r^{⋆}L_{s}(v^{⋆}{)}_{end}.$$

For each dynamic phase, the target was mapped from the reference vessel coordinate to the phase-specific centerline using branch-position correspondence along the main branch. This yielded a phase-specific target interval $\Gamma_{t}(v^{⋆})=[r_{t,min},r_{t,max}]$. In the physician-facing software interface, the operator selected the intended target by clicking the target vessel and target position on the standard reference template according to procedural judgment. The system then recorded the corresponding vessel name and relative position on the reference map for subsequent dynamic-phase mapping. In this way, the robotic target was represented not as an image-space point but as a vessel-–coordinate interval embedded in the dynamic coronary map. This representation decoupled robotic targeting from raw image coordinates and allowed a physician-defined target to remain interpretable across dynamic cardiac phases within a unified vessel-coordinate control framework.

### 2.6. Live Non-Contrast Guidewire State Estimation

During robotic guidewire navigation, fluoroscopy was acquired without coronary contrast injection. A guidewire-stage perception model segmented both guiding catheter and guidewire structures. The resulting masks were post-processed to suppress irrelevant image regions, retain major connected components, close small gaps, and reconnect fragmented guidewire components. The processed segmentation was skeletonized to yield two complementary live representations: a weighted catheter–guidewire skeleton $S_{live}$ and an ordered guidewire path $Q=\{q_{1},\ldots ,q_{m}\}$.

Cardiac phase was estimated by comparing the live catheter–guidewire skeleton with each stored vessel–catheter template. The phase score was defined as

(3) $$s_{t}=\sum_{x}S_{live}(x)M_{t}(x),$$

and the selected phase was

(4) $$t^{⋆}=arg\underset{t}{max}s_{t}.$$

This phase-matching step used both catheter geometry and guidewire trajectory, because the live skeleton encoded both structures and the stored template preserved both vessel and catheter information. As an overlap-based operational estimate, this step may be affected by respiratory drift, catheter engagement changes, or unintended device motion that is not purely cardiac phase-related; these factors were treated as robustness limitations of the current preclinical implementation.

After phase selection, the ordered guidewire path was assigned to a named vessel branch in the selected dynamic map $G_{t^{⋆}}$. The similarity score combined average spatial deviation, maximum spatial deviation, and distal direction mismatch:

(5) $$Sim(Q^{\prime },v)=\lambda_{1}\frac{\bar{d}(Q^{\prime },P_{t^{⋆}}(v))}{10}+\lambda_{2}\frac{d_{max}(Q^{\prime },P_{t^{⋆}}(v))}{20}+\lambda_{3}\Delta \theta (Q^{\prime },P_{t^{⋆}}(v)),$$

where $\lambda_{1}$, $\lambda_{2}$, and $\lambda_{3}$ are the weighting coefficients for mean spatial deviation, maximum spatial deviation, and distal direction mismatch, respectively. In the current implementation, these coefficients were empirically selected during system development and should not be interpreted as globally optimized weights across all coronary anatomies. The current vessel was then estimated as

(6) $$v_{cur}=arg\underset{v}{min}Sim(Q^{\prime },v).$$

The guidewire tip $q_{tip}$ was projected onto the selected centerline according to

(7) $$i^{⋆}=arg\underset{i}{min}{∥P_{t^{⋆}}(v_{cur})[i]-q_{tip}∥}_{2}.$$

**Figure 5:**
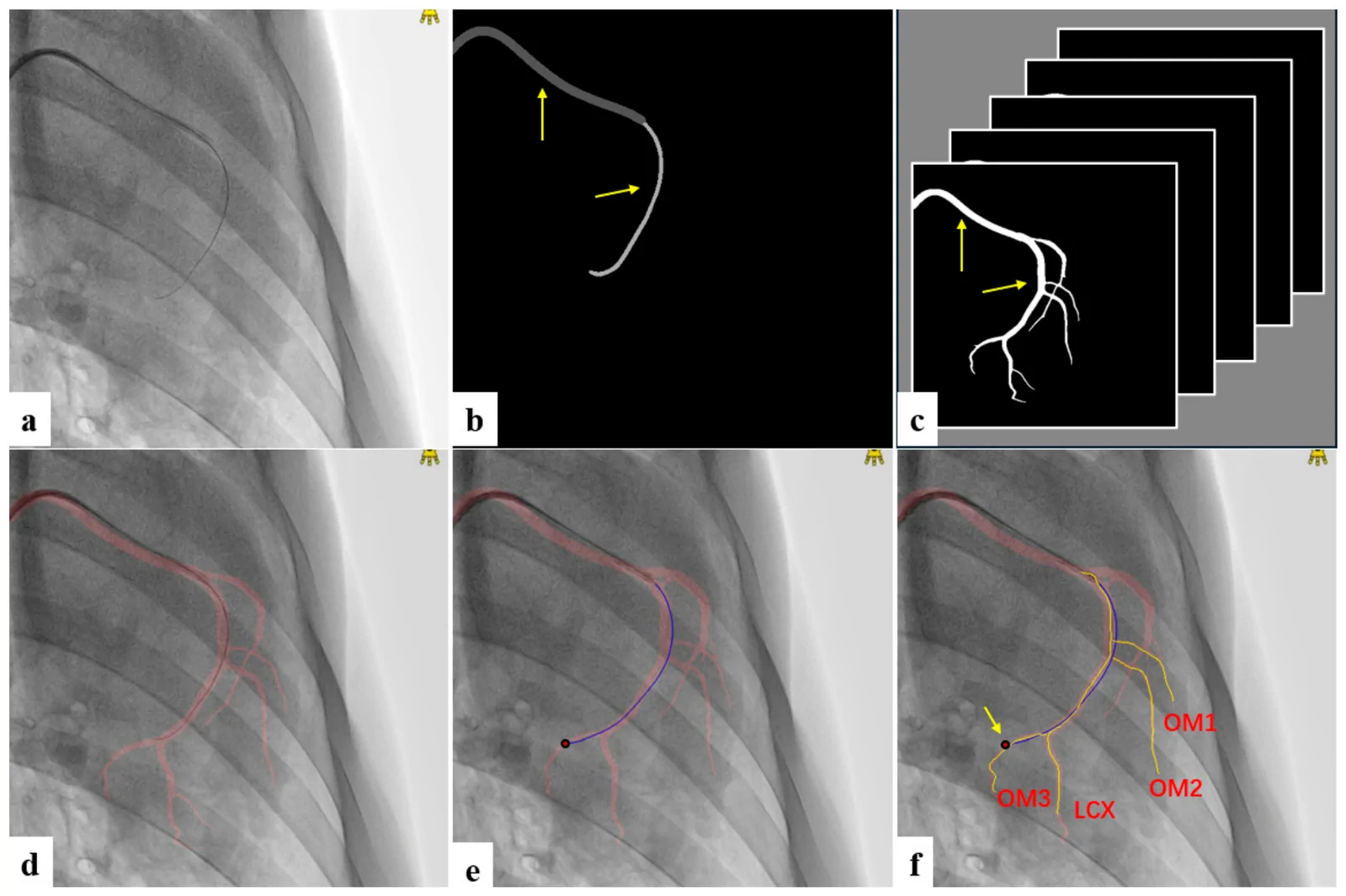
Live non-contrast guidewire localization and dynamic map matching. (**a**) A live fluoroscopic frame is acquired during guidewire manipulation without contrast injection. (**b**) The catheter–guidewire segmentation model separates guiding catheter and guidewire labels, followed by post-processing and skeletonization. (**c**) The contrast-stage vessel–catheter template library provides phase-indexed masks for matching. (**d**) Cardiac phase is selected by maximizing overlap between the live catheter–guidewire skeleton and each stored vessel–catheter template. (**e**) The ordered guidewire path is used to identify the distal tip. (**f**) The guidewire path is matched to the most similar named vessel branch in the selected dynamic skeleton map, and the tip is projected onto that centerline to obtain a vessel name and arc-length coordinate.

The resulting live robotic state consisted of the selected phase $t^{⋆}$, current vessel $v_{cur}$, tip arc-length coordinate $L_{t^{⋆}}(v_{cur})[i^{⋆}]$, and centerline projection error. This nearest-centerline projection was used as a real-time operational approximation. It may become less stable near bifurcations, when centerline spacing is non-uniform, or when the guidewire tip is poorly visible because of overlap, foreshortening, or out-of-field motion. These cases define important boundary conditions for future robustness analysis. Figure 5 summarizes this live non-contrast state-estimation pipeline.

### 2.7. Robot-Command Generation

The robotic action module compared the current vessel-coordinate state with the phase-specific target interval. If the guidewire tip was in the target vessel and within the target interval, the controller issued a stop command. If the tip was in the target vessel but proximal to the interval, the controller advanced the guidewire; if distal to the interval, it retreated. If the guidewire entered a non-target side branch, the controller issued a retreat command. If the target was a side branch and the guidewire remained in the main branch, the controller advanced toward the branch origin and added rotation-assisted advancement when guidewire direction was inconsistent with target-branch direction. Directional consistency was implemented as a categorical comparison rather than as a continuous angular threshold. The distal guidewire direction and target-branch direction were each estimated from three-point bending geometry and categorized as left, right, or straight. When the guidewire approached the target branch but the distal guidewire category did not match the target-branch category, the controller issued a combined axial-and-rotational command. In the implemented prototype, this typically consisted of a short forward step of 3–5 mm combined with an empirically specified rotational command; wrong-branch or hidden-branch retreat commands similarly combined axial retreat with rotation when appropriate. Thus, rotation-assisted behavior was implemented as an expert-inspired exploratory maneuver rather than as an optimized continuous angular-control law.

**Figure 6:**
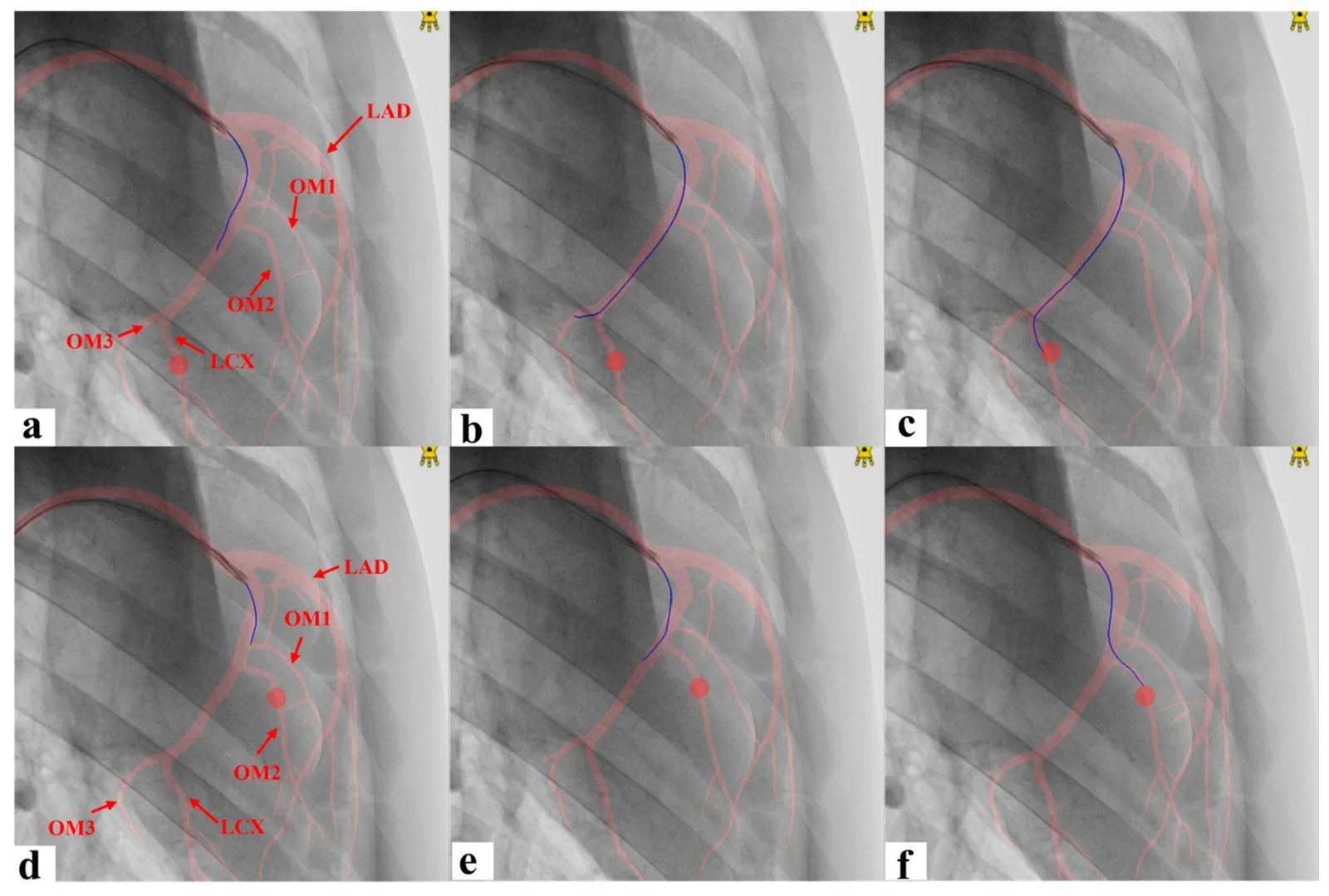
Robot action state machine for guidewire navigation. (**a**–**c**) Robot action logic when the target vessel is the main branch: the system advances when the guidewire is proximal to the target interval, stops when the tip is within the target interval, and retreats if the tip passes the target. (**d**–**f**) Robot action logic when the target vessel is a side branch: the system advances along the main branch toward the target bifurcation, adds rotation-assisted advancement when guidewire direction is inconsistent with the target-branch direction, and retreats when the guidewire enters a wrong branch or overshoots the target. In each panel, the red shadow denotes the selected vessel segmentation, the blue curve denotes the detected guidewire, and the red circle denotes the target position. Additional safety states include hidden or unmodeled branch detection and stop at target arrival.

Hidden or unmodeled branch entry was handled conservatively by retreat based on centerline projection error. In the current implementation, hidden-branch retreat was triggered when the distance between the guidewire tip and the matched centerline exceeded an empirical threshold of 20 pixels. This value was selected during system development as a practical compromise between false-positive retreat triggers and false-negative missed hidden-branch entries. The rule was intended as a safety-oriented heuristic for cases in which the visible guidewire deviated from the selected mapped centerline, suggesting entry into a small or unmodeled branch. The action logic used for these control decisions is illustrated in Figure 6.

### 2.8. Evaluation Definitions and Statistical Reporting

Segmentation performance was evaluated on held-out test images using Dice similarity, centerline distance, centerline Hausdorff distance, and distal endpoint hit rate where applicable. The image-level unit for each segmentation task is reported in Table 2 and Table 3. Vessel, catheter, and guidewire test-set denominators differed because the three segmentation tasks used task-specific annotated images rather than a single shared image set.

Cardiac phase selection was evaluated on live animal-experiment fluoroscopy frames. Expert reference labels for phase consistency and vessel identity were assigned by two on-site physician experts, one junior and one senior, who independently reviewed the fluoroscopic sequences with reference to the available angiographic and dynamic map context. For phase labeling, the experts visually compared each live non-contrast fluoroscopic frame with the stored contrast-phase dynamic sequence and selected the closest matching phase by considering guiding catheter position, guidewire trajectory, cardiac silhouette or boundary motion, and overall anatomical alignment. Because the coronary lumen was not visible during non-contrast navigation and a live frame did not necessarily correspond exactly to one stored contrast phase, the expert phase label was interpreted as a best-matching phase estimate rather than an absolute frame-level ground truth. The two experts showed more than 95% agreement for guidewire/vessel-identity interpretation, whereas exact phase-label agreement was approximately 80%. This difference reflected the nature of the two annotation tasks: vessel identity could usually be inferred from the visible guidewire trajectory and its branch relationship, whereas exact phase labeling required matching a non-contrast frame to a discrete set of stored contrast-phase templates despite continuous cardiac motion. Most phase-label disagreements occurred when two adjacent stored phases both provided visually plausible matches to the same live frame. Disagreements were resolved by discussion, with the senior expert providing the final adjudicated reference label. A phase prediction was counted as correct when the selected dynamic template index was within ±1 frame of the expert reference index. This tolerance was used because adjacent fluoroscopic frames often represent closely neighboring cardiac-motion states and because the clinical need during non-contrast navigation is approximate phase-consistent guidance rather than exact frame identity. Vessel assignment was counted as correct when the inferred named vessel matched the expert vessel label. Low-confidence expert annotations were excluded from both evaluations.

Robot-command performance was evaluated at both the scenario-episode level and the command-decision level. A scenario episode denotes one target navigation or safety scenario, such as main-branch target navigation, side-branch target navigation, wrong-branch retreat, or hidden-branch retreat. A command-level decision denotes one robot action generated within an episode. Correct-command rate and unsafe-command rate were calculated over command-level decisions rather than over scenario episodes. Accordingly, these percentages describe controller behavior during sequential robotic guidance and should not be interpreted as if each command were a fully independent clinical event. A correct command was defined as a command that matched the physician-supervised reference action for the current vessel-coordinate state and target relationship. For reference-action labeling, physicians selected the relevant dynamic phase, overlaid the corresponding dynamic map, visually assessed the relative position of the guidewire and vessel segmentation, and assigned the appropriate action category, including advance, retreat, stop, or rotation-assisted advance when applicable. An unsafe command was defined as a command likely to worsen the procedural state, including advancing in a wrong branch, advancing beyond the target interval, or failing to retreat when hidden or unmodeled branch entry was suspected; this metric denotes deviation from a safety-relevant reference action rather than observed vessel injury. For percentage outcomes with known denominators, integer counts and Wilson 95% confidence intervals were reported.

## 3. Results

The results are reported according to the evaluation units defined in the study design. Image-level results describe segmentation performance on task-specific test sets. Frame-level results describe phase selection and vessel assignment on real-time fluoroscopy frames from continuous animal-experiment navigation sequences. Scenario-level episodes describe procedural navigation contexts, and command-level decisions describe sequential robot actions within those episodes. These denominators should therefore be interpreted as complementary evidence for preclinical workflow feasibility rather than as a single pooled sample size or as independent clinical-event counts.

### 3.1. Study Data and Perception Performance

All quantitative results were derived from three physician-supervised animal experiments, whereas multiple earlier animal experiments were used for workflow refinement. The segmentation dataset included vessel, guidewire, and catheter image sets with training, validation, and test splits as shown in Table 2. These segmentation datasets were used to verify that the perception front end could support the downstream robotic workflow; by themselves, they do not constitute downstream robotic performance evaluation.

Perception performance is summarized in Table 3. On the task-specific test splits, the vessel, catheter, and guidewire models achieved mean Dice values of 90.7%, 92.6%, and 89.0%, respectively. Mean centerline distances were below one pixel for all three tasks, and the distal endpoint hit rates were 92.8% for catheters and 96.2% for guidewires using a five-pixel threshold. The test-image counts differ across tasks because each segmentation model was evaluated on the corresponding available annotated dataset rather than on an identical shared image set.

### 3.2. Vessel-Coordinate State Estimation

The dynamic map state-estimation results are reported in Table 4. On 623 real-time fluoroscopy frames, phase selection based on overlap between the live catheter–guidewire skeleton and stored vessel–catheter templates achieved 589/623 correct predictions, corresponding to 94.6% accuracy (95% CI, 92.5–96.1%). Because adjacent frames represented neighboring cardiac-motion states, phase selection was evaluated using a pragmatic ±1 frame tolerance criterion rather than exact frame identity alone. Vessel assignment achieved 575/623 correct predictions, corresponding to 92.3% accuracy (95% CI, 89.9–94.1%). These results support the feasibility of converting non-contrast fluoroscopy into a phase-aware, vessel-aware control state for robotic PCI.

For Table 4, phase selection was counted as correct when the selected template index was within ±1 frame of the expert reference index. Vessel assignment was counted as correct when the inferred vessel label matched the expert vessel label. Low-confidence expert annotations were excluded from both evaluations. This evaluation criterion reflects the practical requirement for phase-consistent guidance during non-contrast navigation, where the vessel lumen is not directly visible and exact framewise coronary anatomy cannot be observed.

### 3.3. Robot-Command Performance

Robot-command performance is summarized in Table 5. The reported dataset included 34 scenario-level episodes across main-branch target navigation, side-branch target navigation, wrong-branch retreat, and hidden-branch retreat scenarios. Because episode counts and command-level decisions are different units, Table 5 reports both the number of episodes and the command-level denominators used for rate calculation. Correct-command rates ranged from 83.6% in main-branch target navigation to 95.6% in wrong-branch retreat, whereas unsafe-command rates were 0% in the main-branch target and wrong-branch retreat categories, 0.8% in side-branch target navigation, and 13.3% in hidden-branch retreat.

**Figure 7:**
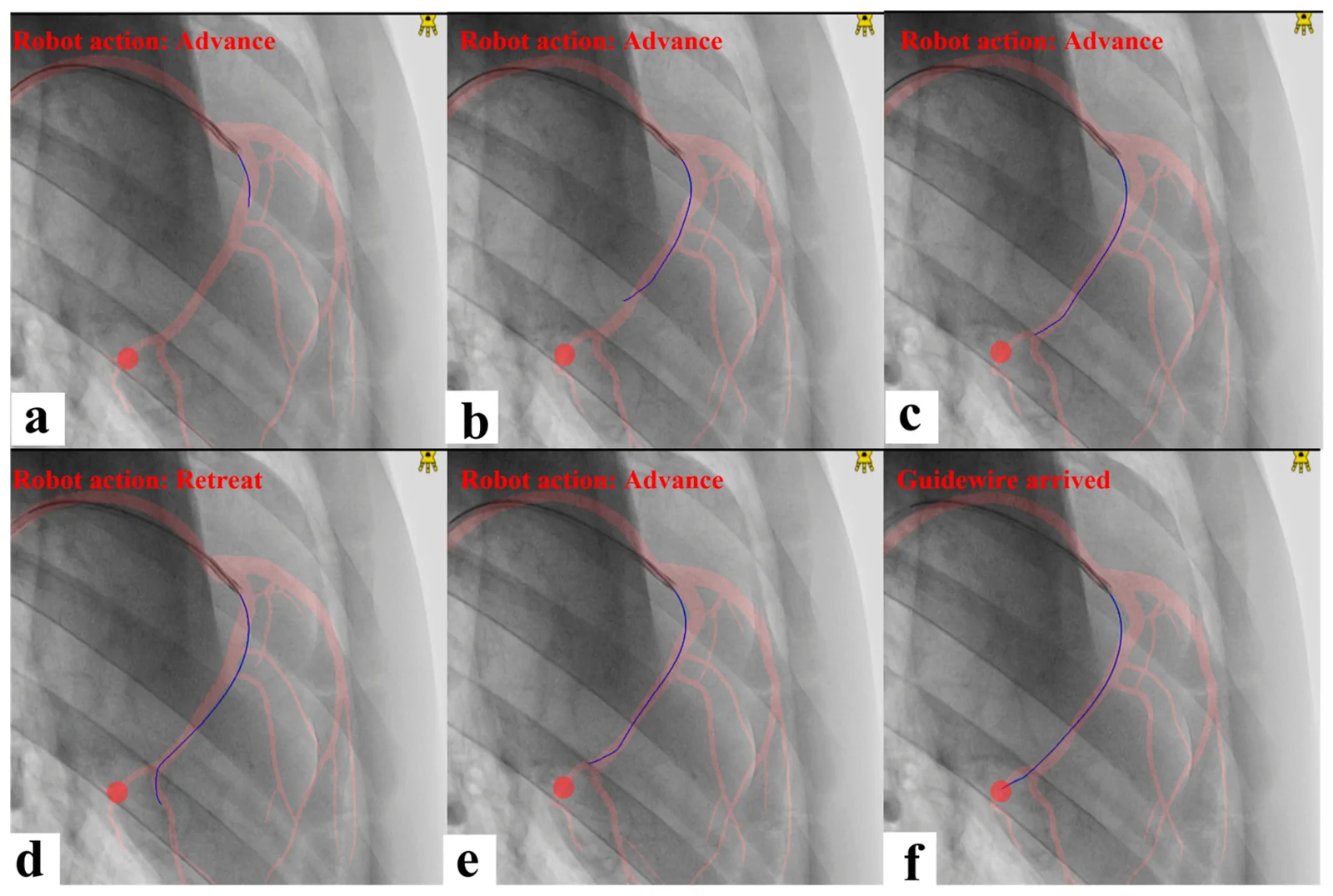
Representative side-branch guidewire navigation by the robot. (**a**) The guidewire is proximal in the main branch and the robot advances. (**b**) The guidewire is in the middle segment of the main branch and the robot continues forward movement. (**c**) The guidewire reaches the bifurcation of the target side branch and the robot advances toward the branch entry. (**d**) The guidewire misses the target side branch and the robot issues a retreat command. (**e**) The guidewire returns to the target bifurcation and the robot advances again. (**f**) The guidewire enters the target side branch and reaches the target position. In each panel, the red shadow denotes the selected vessel segmentation, the blue curve denotes the detected guidewire, and the red circle denotes the target position.

The hidden-branch category was the most challenging scenario. Small side branches are common in coronary anatomy and may be incompletely represented in a named dynamic map, particularly when contrast visibility is limited or branch skeletons are weak. In these episodes, incomplete or weakly represented branch structure could reduce the reliability of vessel-coordinate interpretation and make conservative retreat triggering more difficult, which helps explain the higher unsafe-command rate observed in this category. A representative side-branch robotic guidewire navigation sequence is shown in Figure 7.

The Episodes column reports scenario-level samples. The command-rate columns report command-level decisions with Wilson 95% confidence intervals and should not be interpreted as proportions of the episode counts.

### 3.4. Real-Time Computational Performance

The proposed workflow was integrated into the live fluoroscopic control loop during animal experiments. The measured computational latency profile is summarized in Table 6.

## 4. Discussion

This study frames AI-driven robotic PCI as a biomedical engineering problem of perception, anatomical state representation, and robot-executable action. Rather than treating fluoroscopic perception and robot manipulation as separate problems, the proposed workflow links them through a vessel-coordinate control framework. The main contribution is therefore not a single segmentation model, matching metric, or control heuristic in isolation, but a live preclinical demonstration that these elements can be connected into a robotic guidewire-navigation workflow. This distinction is important for robotic coronary intervention. A robot can execute motion, but meaningful intervention requires anatomical context, target context, and safety-aware state interpretation.

From a translational bioengineering perspective, the main contribution is the integration of four elements within one preclinical system: contrast-stage dynamic coronary mapping, live non-contrast device perception, vessel-coordinate state estimation, and robotic guidewire command generation. This integration differentiates the work from physician-facing roadmapping overlays alone. For example, catheter-tip tracking and dynamic overlay methods can improve human navigation [13], but they do not directly provide the state abstraction required for robot control. The central methodological difference in the present study is therefore not only dynamic anatomical visualization, but the conversion of fluoroscopic perception into a robot-executable vessel-coordinate action state. In the present framework, the guidewire is localized not only in image space but also in a dynamic named vessel-coordinate system linked to robot actions.

The work is also distinct from isolated perception studies. While prior guidewire segmentation and localization studies have advanced fluoroscopic device analysis [14,15,16,17], they do not by themselves solve the action-selection problem in robotic PCI. Similarly, reinforcement-learning and AI-agent work for endovascular guidewire navigation has provided valuable proof-of-concept control insights [18,19,20], but preclinical guidewire control on a PCI robot introduces additional constraints related to moving coronary anatomy, non-contrast navigation, catheter geometry, and physician-defined procedural targets. The present study contributes an interpretable systems methodology tailored to those constraints. Component-level benchmarks and ablations remain important for future optimization, but the present evidence is aimed at the feasibility of integrated AI-robotic PCI workflow execution.

The experimental findings support the feasibility of this approach. Segmentation performance was sufficient to support stable centerline-based representations. Phase selection and vessel assignment achieved high frame-level accuracy in 623 real-time fluoroscopy frames acquired from continuous animal-experiment navigation sequences. Robot-command results further showed strong correct-command rates across target navigation and safety scenarios, although performance varied by scenario complexity and the hidden-branch category remained the most challenging. The hidden-branch results identify a meaningful current safety boundary: small or weakly modeled branches can disrupt the relationship between the visible guidewire and the dynamic map, and future versions should incorporate stronger branch-ambiguity detection and safety-gated retreat logic. Taken together, these findings support the use of vessel-coordinate control as a practical abstraction for AI-driven robotic guidewire navigation.

Several limitations should be acknowledged. First, the study is preclinical and focused on guidewire-navigation subtasks rather than complete PCI. Second, although animal experiments provide physiologically relevant cardiac motion and procedural workflow constraints, they do not fully reproduce diseased human coronary anatomy, lesion morphology, calcification, lesion-crossing mechanics, or the full range of human coronary tortuosity. Human translation will require additional training data, platform-specific validation, and prospective safety testing. At the same time, the basic guidewire manipulation primitives used by vascular interventional robots—advancement, retreat, and rotation—are broadly relevant across robotic device platforms even when mechanical implementation details differ.

Third, the sample size should be interpreted according to the evaluation layer. The segmentation test sets contain image-level samples, the 623 fluoroscopy frames support frame-level state-estimation analysis, and the 34 scenario-level episodes support the early evaluation of navigation contexts and associated command sequences. These data are appropriate for a preclinical feasibility study of an integrated workflow, but they do not constitute a large-scale clinical performance study and should not be interpreted as proof of superiority over manual or teleoperated robotic PCI. Larger independent task-level datasets, additional animal models, human angiographic data, and controlled comparisons with manual robotic operation are needed to evaluate generalization and comparative advantage.

Fourth, the dynamic map library should be interpreted as a guidance-oriented phase-conditioned representation rather than as a fully independently validated frame-by-frame anatomical correspondence benchmark. Exact dynamic correspondence is difficult to define during non-contrast navigation because the coronary lumen is not visible, and physicians themselves rely on approximate anatomical memory and phase-consistent inference during fluoroscopic guidewire manipulation. Fifth, the controller is rule-based and does not explicitly propagate probabilistic uncertainty from segmentation, phase matching, or vessel assignment to command generation. Sixth, the reported frame-level and command-level evaluations may include temporal dependence within the same sequences. Command-level percentages describe sequential controller behavior within episodes and are not equivalent to fully independent clinical-event counts.

Additional limitations concern robustness and generalization. The workflow was field-iterated in a specific preclinical robotic environment, which is a strength for translational feasibility but also means that external validation across additional imaging systems, robotic platforms, anatomies, and guidewire types remains necessary. Respiratory drift, catheter engagement shifts, changing device geometry, map obsolescence over time, guidewire-tip invisibility, and bifurcation-adjacent projection ambiguity were not fully characterized in the current study. The navigation tasks used physician-defined targets that remained fixed during each evaluated episode; intra-procedural dynamic retargeting was not evaluated. Full robotic hardware disclosure, exhaustive ablation of individual algorithmic components, and full procedural automation remain outside the scope of the present manuscript.

The current framework also separates task-level guidewire command generation from low-level robotic trajectory optimization. The action module generates clinically interpretable guidewire commands, including advancement, retreat, stop, and rotation-assisted advancement, based on vessel-coordinate state and target relationship. Smooth actuator-level execution, velocity-profile design, and jerk-limited trajectory planning remain important robotic-control topics. Minimum-jerk trajectory planning with piecewise quintic polynomial interpolation has been studied for smooth motion generation in translational parallel manipulators [21]. Although that setting differs from flexible intracoronary guidewire manipulation, similar motion-smoothing concepts may be useful in future execution-layer optimization for robotic PCI devices.

These limitations also define the translational next steps. Future comparative studies should evaluate whether AI-driven robotic PCI can reduce repeated contrast use, fluoroscopy time, radiation burden, navigation time, and operator workload relative to manual robotic operation, while also characterizing failure modes and safety boundaries. Future technical work should also evaluate temporal consistency, component-level ablation, template-composition alternatives, uncertainty-aware control, device-specific adaptation, and smoother low-level execution profiles. Within the scope of the current preclinical study, however, the results support the feasibility of an integrated AI-driven robotic PCI workflow built around dynamic vessel-coordinate control.

## 5. Conclusions

We developed and evaluated a preclinical biomedical engineering framework for AI-driven robotic PCI guidewire control. The system combines contrast-stage dynamic coronary map construction, live non-contrast fluoroscopic state estimation, vessel-coordinate target representation, and robot-command generation on an integrated robotic PCI platform. The results support the feasibility of translating fluoroscopic perception into robot-executable coronary guidewire actions and provide a foundation for larger independent task-level studies, broader preclinical validation, and future comparative translational studies.

## Figures and Tables

**Table 1:** AI + robot task decomposition for PCI guidewire navigation.

Layer	Input	Output	Robot Relevance	Metric
AI perception	Angiography/fluoroscopy	Vessel, catheter, wire masks	Provides visual understanding	Dice, tip error
Dynamic vessel memory	Contrast frames	Phase-indexed named maps	Provides robot navigation map	Branch accuracy
State estimation	Live skeleton + map library	Vessel, phase, tip arc length	Converts image to robot state	Phase/vessel accuracy
Action module	State + target	Robot command	Executes PCI subtask	Command accuracy

**Table 2:** Dataset and annotation summary for segmentation model development.

Split	Vessel Images	Guidewire Images	Catheter Images
Training	6269	3857	4796
Validation	1567	964	1199
Test	957	537	667

**Table 3:** Perception metrics on the test split listed in Table 2.

Task	Model	Test Images	Mean Dice	Mean Centerline Distance (Pixels)	Centerline Hausdorff Distance (Pixels)	Tip Hit Rate
Vessel	nnU-Net	957	90.7%	0.92	4.26	NA
Catheter	Attention U-Net	667	92.6%	0.69	3.13	92.8%
Guidewire	nnU-Net	537	89.0%	0.64	2.97	96.2%

**Table 4:** State-estimation metrics from real-time animal-experiment fluoroscopy frames.

Task	Evaluation Set	Correct/Total	Accuracy	95% CI
Phase selection	623 frames	589/623	94.6%	92.5–96.1%
Vessel assignment	623 frames	575/623	92.3%	89.9–94.1%

**Table 5:** Robot-command metrics.

Scenario	Episodes	Command Decisions	Correct Commands	Unsafe Commands	Correct Command Rate (95% CI)	Unsafe-Command Rate (95% CI)	Time to Target
Main-branch target	9	140	117	0	83.6% (76.6%, 88.7%)	0% (0, 2.7%)	78.7 s
Side-branch target	10	242	215	2	88.8% (84.3%, 92.2%)	0.8% (0.23%, 2.96%)	172.6 s
Wrong-branch retreat	8	23	22	0	95.6% (79.0%, 99.2%)	0% (0, 14.3%)	13.2 s
Hidden-branch retreat	7	15	13	2	86.7% (62.1%, 96.3%)	13.3% (3.7%, 37.9%)	6.8 s

**Table 6:** Computational latency of the real-time perception-to-action workflow.

Processing Component	Mean ± SD (s/Frame)	Range (s/Frame)
Segmentation inference	0.182 ± 0.014	0.157–0.215
Phase matching	0.030 ± 0.003	0.027–0.036
Vessel assignment	0.001 ± 0.001	0.001–0.006
Action generation	0.019 ± 0.002	0.014–0.029
Total frame-to-command latency	0.232 ± 0.015	0.200–0.270

## Data Availability

Correspondence and requests for materials and data should be addressed to the corresponding author. The datasets generated and/or analyzed during the current study are available from the corresponding author on reasonable request, subject to institutional approvals, animal-welfare ethics requirements, data-use agreements, and restrictions related to proprietary robotic-platform information.
